# Identifying patterns of potentially preventable hospitalisations in people living with dementia

**DOI:** 10.1186/s12913-022-08195-9

**Published:** 2022-06-20

**Authors:** Lidia Engel, Kerry Hwang, Anita Panayiotou, Jennifer J. Watts, Cathrine Mihalopoulos, Jeromey Temple, Frances Batchelor

**Affiliations:** 1 School of Public Health and Preventive Medicine, Monash University, Level 4, 553 St. Kilda Road, Melbourne, VIC 3004 Australia; 2 Deakin University, Burwood, Australia; 3 National Ageing Research Institute, Parkville, Australia; 4 The University of Melbourne, Parkville, Australia; 5 Safer Care Victoria, Melbourne, Australia

**Keywords:** Dementia, Hospitalization, Primary health care, Ambulatory care sensitive conditions, Quality of health care, Australia

## Abstract

**Background:**

Older Australians make up 46% of all potentially preventable hospitalisations (PPHs) and people living with dementia are at significantly greater risk. While policy reforms aim to reduce PPHs, there is currently little evidence available on what drives this, especially for people living with dementia. This study examines patterns of PPHs in people living with dementia to inform service delivery and the development of evidence-based interventions.

**Methods:**

We used the Victorian Admitted Episodes Dataset from Victoria, Australia, to extract data for people aged 50 and over with a diagnosis of dementia between 2015 and 2016. Potentially avoidable admissions, known as ambulatory care sensitive conditions (ACSCs), were identified. The chi-square test was used to detect differences between admissions for ACSCs and non-ACSCs by demographic, geographical, and administrative factors. Predictors of ACSCs admissions were analysed using univariate and multiple logistic regression.

**Results:**

Of the 8156 hospital records, there were 3884 (48%) ACSCs admissions, of which admissions for urinary tract infections accounted for 31%, followed by diabetes complications (21%). Mean bed-days were 8.26 for non-ACSCs compared with 9.74 for ACSCs (*p* ≤ 0.001). There were no differences between admissions for ACSCs and non-ACSCs by sex, marital status, region (rural vs metro), and admission source (private accommodation vs residential facility). Culture and language predicted ASCS admission rates in the univariate regression analyses, with ACSC admission rates increasing by 20 and 29% if English was not the preferred language or if an interpreter was required, respectively. Results from the multiple regression analysis confirmed that language was a significant predictor of ACSC admission rates.

**Conclusions:**

Improved primary health care may help to reduce the most common causes of PPHs for people living with dementia, particularly for those from culturally and linguistically diverse backgrounds.

**Supplementary Information:**

The online version contains supplementary material available at 10.1186/s12913-022-08195-9.

## Background

While many hospital admissions are necessary and unavoidable, some individuals may require hospital care for conditions that could have been effectively managed and treated in the community. Such hospital admissions are referred to as Potentially Preventable Hospitalisations (PPHs) and are often used as a proxy measure of primary care effectiveness, with higher rates suggesting lack of timely, accessible and adequate primary care [1]. Although PPHs are difficult to define and measure, a commonly used proxy measure for PPHs is admissions for ambulatory care sensitive conditions (ACSCs) [2], which can be divided into three main categories: 1) vaccine-preventable conditions (e.g. influenza, pneumococcal infection); 2) acute treatable conditions (e.g. dehydration, gastroenteritis); and 3) chronic manageable conditions (e.g. asthma, diabetes complications) [3]. While hospital admissions for vaccine-preventable ACSCs are deemed preventable, admissions for acute ACSCs may not be preventable but, in theory, should not result in hospitalisation if primary care is delivered in a timely and effective manner, whereas admissions for chronic conditions may be preventable in some instances through behaviour modification and lifestyle change.

Compared to age-matched peers, people living with dementia are at significantly greater risk of admissions for ACSCs [4–6]. In a US study, it was found that admissions for ACSCs were 78% more common in people living with dementia, accounting for 28% of all hospitalisations compared with only 19% among those without dementia [4]. Potential reasons for high rates of admissions for ACSCs could include that people with dementia might be more prone to infections due to reduced mobility or inadequate fluid intake and are less likely to seek help because of diminished recognition of symptoms or inability to communicate symptoms [7]. Often, symptoms can cause secondary behavioural or psychological symptoms of delirium, which could be linked mistakenly to dementia rather than underlying health conditions, leading to delayed diagnosis of conditions that could have been controlled in primary care [7]. Another US-based study has shown that the likelihood of admission for ACSCs among people with dementia was higher in rural areas compared with metropolitan residents, indicating that rural areas may face barriers in accessing timely and effective primary care [8]. Other reasons that have been identified as predictors of PPHs outside of dementia include socioeconomic characteristics, lifestyle factors, cultural and health service system barriers [1, 9–11]. However, the exact factors leading to PPHs for people living with dementia are currently unknown, which means that interventions cannot be appropriately developed and implemented [11].

Reducing PPHs among older Australians has been a key government priority, particularly for those living with dementia [12]. A report from 2017 to 2018 has shown that 7 % of all hospitalisations in Australia could have been prevented if conditions were managed earlier through access to primary or preventative care [13]. Older Australians aged 65 years and above make up 46% of all PPHs, and rates increase with increasing geographic remoteness and socioeconomic disadvantage [14]. However, to date, PPHs among people living with dementia in Australia have not been explored. In 2018-2019, approximately 23,200 hospitalisations were due to dementia as the principal diagnosis or the main reason for admission [15]. An increasing body of evidence suggests that people living with dementia are at greater risk of poor outcomes during hospitalisation, often leading to adverse events, readmission, transfer to permanent residential care, and mortality [16, 17]. Seventy four percent of all episodes of care for people living with dementia result in complications or comorbidities, compared to 45% for older people without dementia [18]. There is a pressing need to explore PPHs to guide the development of evidence-based interventions that improve access to and the quality of healthcare for people living with dementia and their carers. Therefore, the aim of this study was to examine patterns of PPHs in people living with dementia to inform service delivery and the development of evidence-based interventions.

## Methods

### Data source

Individual hospital separation data were obtained from the Victorian Admitted Episodes Dataset (VAED). Victoria is the second largest state in Australia, with a population of 6.7 million people [19]. There are an estimated 472,000 people living with all forms of dementia in Australia, with 120,900 people residing in Victoria [20]. The VAED is a minimum dataset containing demographic, clinical and administrative data for every admitted episode of care occurring in all Victorian acute hospitals, both public and private. Given that hospitals are regulated by the respective States and Territories in Australia, the VAED is managed by the Victorian Department of Health to support health service planning, policy formulation, epidemiological research and public hospital funding under the case-mix system [21]. Since the data collection is subject to regular audits, the diagnosis and procedure coding of the VAED is considered to be of good-to-excellent quality [22]. Ethics approval for this project was obtained from the Deakin University Human Research Ethics Committee (2019-022). All methods were carried out in accordance with the Australian National Statement on Ethical Conduct in Human Research.

### Study population

Hospital separation data were obtained between 30 June 2015 – 1 July 2016 for all people aged 50 and over, where dementia was documented as a principal or additional diagnosis based on the following ICD-10-AM (International Classification of Diseases, 10th revision, Australian modification) 9th edition codes: F00, F01, F02, F03, F051, G30, G31. The VAED allows up to 40 diagnosis and procedure fields based on ICD-10 AM. Hospital separation refers to the process by which an admitted patient completes an episode of care by being discharged, dying, transferring to another hospital or changing type of care. Hospital episodes with admission source being a transfer from another hospital or a type change admission (i.e., statistical admission) were excluded in order to reduce multiple counting of hospitalisation episodes relating to the same event. We selected age 50 rather than 65 as the threshold, reflecting the eligibility criteria for accessing aged care services of older Aboriginal and Torres Strait Islander population in Australia, where many conditions and comorbidities are early onset in this population [23].

### Outcome measures

The primary outcome for this study was the number of hospital admissions for ACSCs among people living with dementia. ACSCs were defined in accordance with the National Healthcare Agreement indicator PI 18-Selected potentially preventable hospitalisations, which in 2018 identified 22 health conditions as ACSCs [3]. Supplementary Table 1 provides the ICD-10-AM codes that classify ACSCs, which were used for the analyses. The VAED records were coded as ACSCs on the basis of diagnosis fields, with some exclusions based on procedure fields. For example, for congestive cardiac failure, some cases were excluded based on certain cardiac procedure codes. While some ICD-10 codes could appear in any diagnosis fields, others were only considered ACSCs if they were noted as a principal diagnosis. All chronic ACSCs were restricted to principal diagnosis, whereas vaccine-preventable and some acute conditions could appear in any of the 40 diagnosis fields. Records were coded as ACSCs for each of the 22 health conditions as well as the three overreaching categories (i.e., vaccine-preventable conditions, acute conditions, and chronic conditions). In addition to this classification of ACSCs, a dichotomous variable was also created, based on the presence or absence of ACSCs (ACSC = 1 if the episode was associated with any ACSC condition and ACSC = 0 otherwise).

The VAED also contains demographic, geographical, and administrative data for every admitted episode. Selected information was requested that was deemed to be important in analysing potential predictors and patterns of ACSCs in people living with dementia based on previous evidence [1, 8, 10, 11]. Demographic characteristics included sex, age, marital status, and indigenous status. Geographical information contained hospital region that distinguishes between rural and metropolitan region of Victoria. Administrative data contained information on the language spoken, whether an interpreter was required and the admission source (i.e., private residence or residential facility). Length of stay was calculated for each separation based on the admission and separation dates provided in the dataset.

### Statistical analyses

Descriptive statistics were used to explore the dataset. Differences between admissions for ACSCs and non-ACSCs were analysed using chi-square test for categorical variables, Fisher’s exact test for variables with small samples, and *t*-test for continuous variables of the two groups. A logistic regression analysis was fitted for each independent variable separately, where ACSCs (0/1) denoted the dependent variable. A multiple logistic regression analysis was then undertaken that included all independent variables into the same model. For all analyses, odds ratios (OR) and 95% confidence intervals (CI) were reported. The likelihood ratio chi-square, which compares the fitted model to an empty model (i.e., a model with no predictors), the pseudo-R-squared, and the AIC (Akaike Information Criterion) and BIC (Bayesian Information Criterion) were explored to assess goodness of fit. Complete data were used for the analyses; listwise deletion was used for handling missing values. A 5% level of statistical significance was used for all analyses. All analyses were undertaken in STATA (version 15, StataCorp).

## Results

Between 2015 and 2016, there were 11,288 hospital admissions recorded in the VAED where dementia was documented as a principal or additional diagnosis. Following the removal of records with admission source being a transfer from another hospital or a type change admission, 8156 records remained, of which 3884 (47.62%) were identified as admissions for ACSCs. Table 1 shows the number of hospital admissions and percentages for conditions that were classified as ACSCs and non-ACSCs.

**Table 1:** ACSCs in people living with dementia in Victoria (2015/2016)

	N	%	% of ACSCs	Mean bed days (SD)
**Non-ACSCs**	4272	52.38	–	8.26 (11.99)
**ACSCs**	3884	47.62	–	9.74 (10.83)
**Chronic**	2186	26.80	56.28	9.68 (10.60)
Asthma	9	0.11	0.23	9.78 (9.86)
Congestive cardiac failure	322	3.95	8.29	9.81 (9.54
Diabetes complications	811	9.94	20.88	8.58 (9.92)
Chronic obstructive pulmonary disease	337	4.13	8.68	8.28 (10.23)
Bronchiectasis	10	0.12	0.26	12.8 (20.69)
Angina	25	0.31	0.64	5.68 (7.56)
Iron deficiency anaemia	112	1.37	2.88	8.16 (6.10)
Hypertension	463	5.68	11.92	12.38 (12.59)
Nutritional deficiencies	88	1.08	2.27	12.52 (10.11)
Rheumatic heart diseases	9	0.11	0.23	16.89 (17.34)
**Acute**	1653	20.27	42.56	9.76 (10.82)
Pneumonia (not vaccine-preventable)	< 5	0.05	0.10	5 (2)
Urinary tract infections, including pyelonephritis	1207	14.80	31.08	9.25 (9.55)
Perforated/bleeding ulcer	19	0.23	0.49	10.63 (8.57)
Cellulitis	207	2.54	5.33	10.95 (11.09)
Pelvic inflammatory disease	< 5	0.01	0.03	8 (n/a)
Ear, nose and throat infections	27	0.33	0.70	12.56 (34.52)
Dental conditions	23	0.28	0.59	11.52 (10.23)
Convulsions and epilepsy	119	1.46	3.06	10.00 (9.70)
Eclampsia	0	0	0	0
Gangrene	46	0.56	1.18	14.78 (14.99)
**Vaccine preventable**	45	0.55	1.16	11.84 (11.99)
Pneumonia and influenza (vaccine-preventable)	23	0.28	0.59	8.26 (5.80)
Other vaccine-preventable conditions	22	0.27	0.57	15.59 (26.42)

*ACSCs* Ambulatory Care Sensitive Conditions

Out of the 3884 ACSCs, most were chronic preventable conditions (56.28%), followed by acute conditions (42.56%) and vaccine preventable conditions (1.16%). The top-five ACSCs hospitalisations included: urinary tract infections (31.08%), diabetes complications (20.88%), hypertension (11.92%), chronic obstructive pulmonary disease (8.68%), and congestive cardiac failure (8.29%). Table 1 also displays the mean bed days of a hospital separation, which were greater for ACSCs (9.74 days ±10.83) compared with admissions of non-ACSCs (8.26 days ±11.99).

Table 2 provides the characteristics of the sample by the presence or absence of ACSCs across demographic, geographical, and administrative factors.

**Table 2:** Characteristics of the sample by ACSCs and non-ACSCs

	Total (***N*** = 8156)^**a**^	Non-ACSCs (***N*** = 4272)	ACSCs (***N*** = 3884)	***p***-value^**b**^
**Sex, N (%)**				
Male	3432 (42.08)	1785 (41.78)	1647 (42.40)	0.570
Female	4724 (57.92)	2487 (58.22)	2237 (57.92)	
**Age groups, N (%)**				
50-84	3666 (44.95)	1905 (44.59)	1761 (45.34)	0.498
85+	4490 (55.05)	2367 (55.41)	2123 (54.66)	
**Marital status, N (%)**				
Never married/ widowed/ divorced/ separated	4330 (53.09)	2285 (53.49)	2045 (52.65)	0.368
Married/de facto	3636 (44.58)	1882 (44.05)	1754 (45.16)	
**Indigenous status**				
Aboriginal or Torres Strait Islander	9 (0.11)	6 (0.14)	3 (0.008)	0.512
Neither aboriginal nor Torres Strait Islander	8075 (99.89)	4229 (99.86)	3846 (99.92)	
**English preferred language, N (%)**				
Yes	6209 (76.13)	3337 (78.11)	2872 (73.94)	≤0.001
No	1947 (23.87)	935 (21.89)	1012 (26.06)	
**Interpreter required, N (%)**				
Yes	1055 (13.48)	470 (11.53)	585 (15.59)	≤0.001
No	6770 (86.47)	3604 (88.42)	3166 (84.36)	
**Hospital region, N (%)**				
Metropolitan	6093 (74.71)	3195 (74.61)	2898 (74.61)	0.855
Rural	2063 (25.29)	1077 (25.21)	986 (25.39)	
**Admission source, N (%)**				
Private residence/accommodation	7608 (93.28)	3976 (93.07)	3632 (93.51)	0.427
Aged care residential facility/ Care bed based program/ Mental health residential facility	548 (6.72)	296 (6.93)	252 (6.49)	
**Bed days, mean (SD)**	8.97 (11.48)	8.26 (12.00)	9.74 (10.83)	≤0.001
**Length of stay type, N (%)**				
Multi-day	6972 (85.48)	3452 (80.81)	3520 (90.63)	≤0.001
Same-day	677 (8.30)	432 (10.11)	245 (6.31)	
Overnight	507 (6.22)	388 (9.08)	119 (3.06)	

*ACSCs* Ambulatory Care Sensitive Conditions

^a^Some numbers do not add-up to the total number because of missing values, patients refused to answer or question unable to be asked

^b^*P*-value based on chi-square test for categorical variables, Fisher’s exact test for variables with small samples, and t-test for continuous variables of the two groups

There was no statistically significant difference between admissions for ACSCs and non-ACSCs by sex, age groups and marital status. Only a hand-full of admissions included in the dataset were for Aboriginal or Torres Strait Islander population. There was no statistically significant difference comparing rural and metropolitan hospital region. A significant difference was detected for preferred language (*p* ≤ 0.001) and whether an interpreter was required (*p* ≤ 0.001), where a greater proportion of people living with dementia reported that English was not their preferred language and that they needed an interpreter when they were admitted to the hospital for ACSCs. There was no difference by admission source, with similar proportions of patients admitted to hospital residing in private residence or accommodation and residential facilities, such as aged care or mental health facilities. Length of stay comprised more multi-day stays for ACSCs than for non-ACSCs, with a mean length of stay of 9.74 days (±10.83) for ACSCs and 8.26 (±12.00) for non-ACSCs.

Table 3 shows the results from the univariate logistic regression analysis, where each independent variable was fitted separately to the model. Sex, age, marital status, hospital region, and admission source did not predict ACSCs. If English was not the preferred language or if an interpreter was required, ACSCs admission rates increased by 20 and 29% respectively. A final model was fitted that included all independent variables (Table 4). The model confirmed the results from the separate logistic regression analysis, indicating an increase in ACSCs for those who reported that English was not their preferred language. Due to high correlation between the variables ‘preferred language’ and ‘interpreter required’ (*r* = 0.77), the variable ‘interpreter required’ was no longer significant in the final model. Running the multiple regression analysis without the variable ‘interpreter required’ did not change the results and ‘preferred language’ still remained a significant predictor of ACSCs.

**Table 3:** Results from the univariate logistic regression analyses

	N	Odds Ratio	SE	***p***-value	95%CI
Sex (ref. Male)	8156	0.97	0.04	0.570	0.89-1.06
Age (ref. 50-84)	8156	0.97	0.04	0.498	0.89-1.06
Marital status (ref. not married)	7966	1.04	0.05	0.368	0.95-1.14
Language (ref. English not preferred)	8156	0.80	0.41	**≤0.001**	0.72-0.88
Interpreter required (ref. yes)	7825	0.71	0.05	**≤0.001**	0.62-0.80
Hospital region (ref. Metro)	8156	1.00	0.05	0.855	0.91-1.12
Admission source (ref. private residence)	8156	0.93	0.08	0.427	0.78-1.11

**Table 4:** Results from the multiple logistic regression analysis (*n* = 7638)

	Odds Ratio	SE	***p***-value	95%CI
Constant	1.30	0.17	0.046	1.00-1.67
Sex (ref. Male)	1.00	0.05	0.989	0.91-1.10
Age (ref. 50-84)	0.97	0.05	0.544	0.86-1.07
Marital status (ref. not married)	1.02	0.05	0.754	0.92-1.12
Language (ref. English not preferred)	0.78	0.07	**0.006**	0.66-0.93
Interpreter required (ref. yes)	0.87	0.92	0.193	0.71-1.07
Hospital region (ref. Metro)	1.07	0.58	0.228	0.96-1.19
Admission source (ref. private residence)	0.91	0.08	0.287	0.76-1.09

LR chi-squared = 37.55 (*p* ≤ 0.001), pseudo R-squared = 0.0036, AIC = 1.382; BIC = -57,679.980

## Discussion

This study examined PPHs in people with dementia in Victoria and found that almost every second hospital admission was for conditions that could have potentially been prevented through the provision of appropriate preventative health interventions and early disease management in primary and community-based care settings. The most commonly recorded ACSCs in people with dementia included urinary tract infections, diabetes complications, and hypertension. A second significant finding of this study from the univariate regression analyses was the detection of admission for ACSCs in people who required an interpreter and who reported that English was not their preferred language. Results from the multiple regression analysis confirmed that language was a significant predictor of ACSC admission rates, indicating potential language barriers in accessing effective primary and community care, which could lead to PPHs.

Our findings align with the report by the Australian Institute of Health and Welfare, which also found that most PPHs in 2017-2018 for people aged 65 and over were for chronic conditions (65%), followed by acute conditions (29%) and vaccine-preventable conditions (12%) [14]. However, compared with older Australians, where the most common reason for hospitalisation for ACSCs included chronic obstructive pulmonary disease, congestive cardiac failure and urinary tract infections [14], in our study sample, we found that 50% of hospital admissions in people with dementia were for urinary tract infections and diabetes complications. When compared with international evidence that focuses on PPHs in people living with dementia, a US study found that three ACSCs accounted for two-third of all potentially preventable admissions that included congestive heart failure, urinary tract infections and bacterial pneumonia [4]. In our sample, the number of ACSCs for pneumonia and influenza (vaccine-preventable) was relatively low, although these are generally the highest in people aged 65 and over and in children under 5 [14]. The reasons for this finding are unclear, which could be due to sampling bias. Pneumonia is of particular concern for older people living in residential care [24], yet the majority of our study sample were admitted into hospital from private residence, which could partially explain the low rates of vaccine preventable conditions and the high number of admissions for diabetes complications and urinary tract infections.

Urinary tract infections are more common in people with dementia due to reduced mobility, inadequate fluid intake, or use of catheters. Early detection of urinary tract infections in people with dementia is particularly challenging because of reduced ability to communicate symptoms [25]. Urinary tract infections often result in behaviour change, including increased confusion and agitation, which are common symptoms of dementia and may therefore result in delayed detection and treatment. Additionally, urinary incontinence is common among people with dementia, making it difficult to detect changes in urinary frequency [25]. To overcome this problem, recent solutions include the use of in-home sensing technologies and machine learning models to detect and predict urinary tract infections in people living with dementia, providing clinical pathways for early interventions [26].

There is also an increased likelihood in the older adult population to develop both dementia and diabetes, with research showing that people with diabetes have a greater rate of decline in cognitive function and an increased risk of dementia [27]. However, diabetes management in people with cognitive impairment and dementia is challenging, where it becomes difficult to achieve the right balance of food, medicine, and physical activity. Similarly to urinary tract infections, some signs and symptoms of poorly managed diabetes are similar to signs and symptoms of dementia [28]. Often, diabetes management is provided by informal family carers who may lack knowledge to optimally care for both dementia and diabetes. It has also been shown that informal carers find that behavioural and psychological symptoms of dementia disrupt the daily diabetes care routine, especially if the person with dementia has limited awareness or understanding of having diabetes or memory loss [29]. Recognising that diabetes care is challenging in people with dementia, specific guidelines have been developed for managing both illnesses simultaneously, which focus on person-centred care as well as providing support and diabetes education for carers [28, 30]. Generally, it is important to recognise that people with dementia have a high prevalence of comorbid medical conditions [31], which requires adaptation in current process of care and service delivery. This should also include the development and evaluation of models to improve dementia literacy in the community, which refers to a person’s knowledge and beliefs regarding dementia [32].

An important finding of this study was the increase in admissions for ACSCs among people with dementia from culturally and linguistically diverse (CALD) backgrounds. Previous literature has shown that cultural attitudes and beliefs, as well as language barriers influence the access to formal services in people with dementia and their carers [33]. The majority of community-dwelling people with dementia rely on the support provided by family carers, but CALD carers may have limited health literacy when making health and care decisions for people with dementia. A qualitative study with carers of people with dementia from CALD groups in Australia revealed that carers face enormous challenges in utilising dementia services, requiring a bilingual service advisor to better support care recipient and caregiver’s needs [34].

Australia relies heavily on immigration and data from 2016 indicates that 37% of older adults aged 65 years and above were born overseas, with the majority born in non-English-speaking countries [35]. Recent population projections demonstrate that by 2046 there will be a move away from a European-born toward an Asian-born dominance in the older Australian population, with rates from Chinese Asia and Southern Asia increasing from 8 to 21% [36]. Although migrants from Chinese and Southeast Asian countries historically have had lower levels of English language proficiency, future migrants from these geographical regions are more likely to have higher English language proficiency than their historic counterparts. However, dementia can result in language reversion and compromise prior gains in English proficiency, where bilingual or multilingual people with dementia revert to their mother tongue, which is often not English [37]. With the rising number of people living with dementia in Australia, projected to increase from 472,000 to 1,076,000 by 2058 [20], ensuring culturally and linguistically appropriate dementia care becomes increasingly vital. However, the provision of evidence-based medical care, social care and aged care services to persons with dementia from CALD backgrounds remains challenging in Australia and internationally [32].

Our study found that the proportion of ACSC hospitalisation admissions in people living with dementia was higher compared with a previous US study [4]. The difference may be due to the classification of ACSCs, as different versions of ACSCs are used around the world, specific to the healthcare system as well as the purpose for which they are being reported. However, this number is similar to PPHs reported among nursing home residents in the US, which found that 44% of hospitalisations were PPHs [38]. While previous studies highlighted that PPHs are common in older people who reside in long-term care facilities [39], we did not observe differences in ACSCs by admission source, which could be due to small numbers of patients referred from a residential facility. In the US, high rates of PPHs among nursing home residents have been linked to structural problems in the system and the misalignment of Medicare (health coverage for older adults) and Medicaid (health coverage for people on low income), where Medicaid programs do not benefit from savings that Medicare accrues from prevented hospitalizations of nursing home residents [40]. Qualitative research from Germany further showed that nursing homes tend to call ambulances and transfer people with dementia to hospitals faster due to fear of legal consequences for not acting appropriately [41].

Compared with the Australian general population that showed higher rates of PPHs with increasing remoteness and indigenous status [14], we did not observe statistically significant differences by hospital region (rural versus metropolitan) and indigenous status. However, only 0.11% of our sample was from the Aboriginal or Torres Strait Islander community. Although Aboriginal and Torres Strait Islander people experience dementia at a rate 3 to 5 times higher than the general Australian population, possibly due to higher rates of chronic diseases, dementia is often less well-recognised by Aboriginal and Torres Strait Islander communities, health workers and service providers for reasons such as geographical barriers in service provision as well as different cultural understandings and possibly lack of education and awareness in communities [42]. In contrast to our findings, PPHs have been found to increase with increasing level of remoteness, indicating barriers in accessing primary and community-care services. An Australian study found that regional primary health practitioners experience many challenges in caring for patients with dementia, including a lack of knowledge in dementia care, diagnostic skills and support pathways; time constraints; and accessing specialist support [43]. Although we did not find differences in ACSC admissions for rural hospitals when compared with metropolitan hospitals, it is also important to note that rurality was defined according to hospital region rather than patients’ place of residence. While it is the patients’ place of residence that is relevant in identifying potential barriers in accessing timely and effective primary care, we were unable to explore PPHs by Local Government Areas (LGAs) due to absence of data.

### Implications for policy and practice

Our study provided a detailed analysis of PPHs in people living with dementia by condition, population subgroups and geography to identify priorities for targeted policy interventions. Our findings have three main policy implications. First, a high proportion of admissions for ACSCs suggest that improvements are needed to provide timely, accessible and adequate primary care to avoid PPHs in people with dementia. Secondly, our study has shown that the presence of dementia may adversely affect the primary care of other conditions, such as urinary tract infections or diabetes management, and interventions are needed to better detect symptoms and person-centred care management. Finally, high number of admissions for ACSCs among people with dementia from CALD backgrounds, suggests that more culturally and linguistically appropriate primary and community care is needed to reduce PPHs for people living with dementia.

To date, the effectiveness of interventions to reduce PPHs has not been demonstrated. A previous review of interventions reported that case management, specialist clinics, care pathways and guidelines, medication reviews, vaccine programmes and hospital at home do not appear to reduce avoidable hospitalisations, but there is some evidence of effectiveness for education, self-management, exercise and rehabilitation, and telemedicine in respiratory and cardiovascular patients [11]. Evidence has also shown that one in five hospitalisations for ACSCs in people with dementia will also result in unplanned 30-day readmissions, suggesting that post-discharge care also may need improvements [44].

Addressing PPHs in people living with dementia needs intervention at multiple levels and include carers in intervention design. Qualitative interviews with informal carers of people with dementia, who experienced a recent hospitalisation due to an ACSC, revealed that carers cannot identify symptoms or need to guess because the person with dementia cannot report symptoms, while at the same time they have pressure to make urgent decisions [45]. Decisions are also often made by carers without consulting the person with dementia even in the initial stages of dementia [41]. Therefore, to address PPHs in people with dementia, recent research has focused on assessing effectiveness of interventions by supporting carers to manage the health needs of the person with dementia [46]. In this context, it is also important to consider health literacy challenges faced by patients and their carers who often do not have a good understanding of where, when and how to manage their health [47]. At the same time, it is important to understand physicians’ views on PPHs, where previous studies have shown that many physicians hold the views that hospitalisations for ACSCs were unavoidable [41, 48]. Therefore, improvements are needed at the system-, physician-, family-, and patient-level to support better access and management of conditions in primary care and community-based care settings.

### Limitations

In interpreting the findings of this study, a few limitations are noteworthy. Although this study made use of routinely collected hospital data and the VAED was found of good-to-excellent coding quality [22], the identification and reporting of dementia is often poor in hospitals and is generally only documented if it contributes to the length of stay in hospital and/or cost of treatment and care [18]. This could have resulted in omission of diagnosis coding of dementia, and hence hospital admission records where dementia was not documented as a principal or additional diagnosis, introducing a potential selection bias. Further, since we only had access to the VAED dataset for 2015/2016, we were unable to explore PPHs trends in people with dementia over time. In the absence of hospital admission data for older adults without dementia, predictors of PPHs in our study need to be interpreted carefully, as we are unable to rule out the absence of the same predictors in older adults without dementia. While previous studies have shown that PPHs may be influenced by socio-economic status, we had no information available about patients’ education or income level to explore this further. It would also be important to explore other characteristics in future studies, including the impact of rurality, health literacy and social connectedness on PPHs. Additionally, some information was only documented for certain care types in the VAED. This included information on carer availability (i.e., whether a person, such as a family member, friend or neighbour has been identified as providing regular on-going care or assistance, not linked to a formal service), which was only reported for care types, such as palliative care programs or geriatric evaluation and management programs. Similarly, Functional Independence Measure (FIM) scores, which provide information about the level of independence of an individual, were only documented for certain care types, like rehabilitation. A previous study has shown that hospitalisations for ACSCs increase dramatically with limitations in activities of daily living (ADL) [49], which we were unable to confirm due to absence of data.

There are also general limitations associated with using ACSCs as a proxy for PPHs, particularly in relation to chronic ACSCs [2]. It is also possible that some hospitalised people with dementia may have received optimum management in primary care but some primary prevention initiatives take a long time to impact on admissions rates. In the absence of a comprehensive data sources available in Australia, we were only able to explore hospital admissions for PPHs without examining the patients’ primary care service usage. Access to other medical and pharmaceutical service use (for example via the Medicare Benefit Schedule (MBS) and Pharmaceutical Benefit Scheme (PBS)) could have provided a clearer overall picture. Such data could have also provided further insights into medication-related PPHs, linking suboptimal processes or care and medication use with subsequent hospitalisation [50]. Therefore, ACSC admission rates can only indicate some reasons for PPHs but there are other factors besides access to primary care that may influence hospitalisation rates and may be difficult to measure. For example, a previous study conducted in Australia found that personal sociodemographic and health characteristics, rather than the supply of general practitioners, were major drivers of PPHs [51]. Further, qualitative research has found that patients generally did not perceive that their admission could have been preventable but factors like support deficits, non-adherence to treatment/medication, mental health and lack of awareness or understanding of condition may have contributed to their admission [52].

## Conclusions

In conclusion, this study has shown that people living with dementia are at greater risk of PPHs, highlighting the need for policy reforms that aim to reduce avoidable hospitalisations. Improved primary health care may help to reduce the most common causes of PPHs for people living with dementia, such as urinary tract infection or diabetes complications, particularly for those from culturally and linguistically diverse backgrounds.

## Supplementary Information

**Additional file 1: Supplementary Table 1.** Ambulatory care sensitive cases and ICD-10-AM codes used in this study.

## Data Availability

The data that support the findings of this study are available from the Centre for Victorian Data Linkage (Victorian Department of Health), but restrictions apply to the availability of these data, which were used under license for the current study, and so are not publicly available. Data can be requested from the Centre for Victorian Data Linkage using the online application form (https://www.health.vic.gov.au/reporting-planning-data/applying-for-data-linkage).
