# Supplementary material for: Identifying patterns of potentially preventable hospitalisations in people living with dementia
Source: BMC Health Serv Res. 2022 Jun 20;22:794. doi: 10.1186/s12913-022-08195-9 (PMC9208182; doi:10.1186/s12913-022-08195-9)
Supplement: Supplementary file 1 — Additional file 1: Supplementary Table 1. Ambulatory care sensitive cases and ICD-10-AM codes used in this study. [file 12913_2022_8195_MOESM1_ESM.docx]

**Supplementary Table 1: Ambulatory care sensitive cases and ICD-10-AM codes used in this study**

| **Category** | **ICD-10-AM codes** | **ICD-10-AM description** | **Additional requirements** |
| --- | --- | --- | --- |
| **Vaccine-preventable conditions** |  |  |  |
| Pneumonia and influenza (vaccine-preventable) | J10 | Influenza due to other identified influenza virus | In any diagnosis.  Exclude people under 2 months. |
|  | J11 | Influenza, virus not identified | In any diagnosis.  Exclude people under 2 months. |
|  | J13 | Pneumonia due to *Streptococcus pneumoniae* | In any diagnosis.  Exclude people under 2 months. |
|  | J14 | Pneumonia due to *Haemophilus influenzae* | In any diagnosis.  Exclude people under 2 months. |
| Other vaccine-preventable conditions | A08.0 | Rotaviral enteritis | In any diagnosis. |
|  | A35 | Other tetanus | In any diagnosis. |
|  | A36 | Diphtheria | In any diagnosis. |
|  | A37 | Whooping cough | In any diagnosis. |
|  | A80 | Acute poliomyelitis | In any diagnosis. |
|  | B01 | Varicella [chicken pox] | In any diagnosis. |
|  | B05 | Measles | In any diagnosis. |
|  | B06 | Rubella [German measles] | In any diagnosis. |
|  | B16.1 | Acute hepatitis B with delta-agent (coinfection) without hepatic coma | In any diagnosis. |
|  | B16.9 | Acute hepatitis B without delta-agent and without hepatic coma | In any diagnosis. |
|  | B18.0 | Chronic viral hepatitis B with delta-agent | In any diagnosis. |
|  | B18.1 | Chronic viral hepatitis B without delta-agent | In any diagnosis. |
|  | B26 | Mumps | In any diagnosis. |
|  | G00.0 | Haemophilus meningitis | In any diagnosis. |
| **Chronic** |  |  |  |
| Asthma | J45 | Asthma | As principal diagnosis.  Exclude children aged less than 4 years. |
|  | J46 | Status asthmaticus | As principal diagnosis.  Exclude children aged less than 4 years. |
| Congestive cardiac failure | I50 | Heart failure | As principal diagnosis.  Exclude cases with the following cardiac procedure codes:  Blocks 600-606, 608-650, 653-657, 660-664, 666, 669-682, 684-691, 693, 705-707, 717 and codes 33172-00[715], 33827-01[733], 34800-00[726], 35412-00[11], 38721-01[733], 90217-02[734], 90215-02[732]. |
|  | I11.0 | Hypertensive heart disease with (congestive) heart failure | As principal diagnosis.  Exclude cases with the following cardiac procedure codes:  Blocks 600-606, 608-650, 653-657, 660-664, 666, 669-682, 684-691, 693, 705-707, 717 and codes 33172-00[715], 33827-01[733], 34800-00[726], 35412-00[11], 38721-01[733], 90217-02[734], 90215-02[732]. |
|  | J81 | Pulmonary oedema | As principal diagnosis.  Exclude cases with the following cardiac procedure codes:  Blocks 600-606, 608-650, 653-657, 660-664, 666, 669-682, 684-691, 693, 705-707, 717 and codes 33172-00[715], 33827-01[733], 34800-00[726], 35412-00[11], 38721-01[733], 90217-02[734], 90215-02[732]. |
| Diabetes complications | E10.0–E10.9 | Type 1 diabetes mellitus | As principal diagnosis. |
|  | E11.0–E11.9 | Type 2 diabetes mellitus | As principal diagnosis. |
|  | E13.0–E13.9 | Other specified diabetes mellitus | As principal diagnosis. |
|  | E14.0–E14.9 | Unspecified diabetes mellitus | As principal diagnosis. |
| COPD | J20 | Acute bronchitis | As principal diagnosis.  Only with additional diagnoses of J41, J42, J43, J44. |
|  | J41 | Simple and mucopurulent chronic bronchitis | As principal diagnosis. |
|  | J42 | Unspecified chronic bronchitis | As principal diagnosis. |
|  | J43 | Emphysema | As principal diagnosis. |
|  | J44 | Other chronic obstructive pulmonary disease | As principal diagnosis. |
| Bronchiectasis | J47 | Bronchiectasis | As principal diagnosis. |
|  | J20 | Acute bronchitis | As principal diagnosis.  Only with additional diagnosis of J47. |
| Angina | I20 | Angina pectoris | As principal diagnosis.  Exclude cases according to the list of procedures excluded from the Congestive cardiac failure category above. |
|  | I24.0 | Coronary thrombosis not resulting in myocardial infarction | As principal diagnosis.  Exclude cases according to the list of procedures excluded from the Congestive cardiac failure category above. |
|  | I24.8 | Other forms of acute ischaemic heart disease | As principal diagnosis.  Exclude cases according to the list of procedures excluded from the Congestive cardiac failure category above. |
|  | I24.9 | Acute ischaemic heart disease, unspecified | As principal diagnosis.  Exclude cases according to the list of procedures excluded from the Congestive cardiac failure category above. |
| Iron deficiency anaemia | D50.1 | Sideropenic dysphagia | As principal diagnosis. |
|  | D50.8 | Other iron deficiency anaemias | As principal diagnosis. |
|  | D50.9 | Iron deficiency anaemia, unspecified | As principal diagnosis. |
| Hypertension | I10 | Essential (primary) hypertension | As principal diagnosis.  Exclude cases with procedure codes according to the list of procedures excluded from the Congestive cardiac failure category above. |
|  | I11.9 | Hypertensive heart disease without (congestive) heart failure | As principal diagnosis.  Exclude cases with procedure codes according to the list of procedures excluded from the Congestive cardiac failure category above. |
| Nutritional deficiencies | E40 | Kwashiorkor | As principal diagnosis. |
|  | E41 | Nutritional marasmus | As principal diagnosis. |
|  | E42 | Marasmic kwashiorkor | As principal diagnosis. |
|  | E43 | Unspecified severe protein-energy malnutrition | As principal diagnosis. |
|  | E55.0 | Rickets, active | As principal diagnosis. |
| Rheumatic heart diseases | I00 | Rheumatic fever without mention of heart involvement | As principal diagnosis. |
|  | I01 | Rheumatic fever with heart involvement | As principal diagnosis. |
|  | I02 | Rheumatic chorea | As principal diagnosis. |
|  | I05 | Rheumatic mitral valve diseases | As principal diagnosis. |
|  | I06 | Rheumatic aortic valve diseases | As principal diagnosis. |
|  | I07 | Rheumatic tricuspid valve diseases | As principal diagnosis. |
|  | I08 | Multiple valve diseases | As principal diagnosis. |
|  | I09 | Other rheumatic heart diseases | As principal diagnosis. |
| **Acute** |  |  |  |
| Pneumonia (not vaccine-preventable) | J15.3 | Pneumonia due to streptococcus, group B | In any diagnosis.  Exclude people under 2 months. |
|  | J15.4 | Pneumonia due to other streptococci | In any diagnosis.  Exclude people under 2 months. |
|  | J15.7 | Pneumonia due to *Mycoplasma pneumoniae* | In any diagnosis.  Exclude people under 2 months. |
|  | J16.0 | Chlamydial pneumonia | In any diagnosis.  Exclude people under 2 months. |
| Urinary tract infections, including pyelonephritis | N10 | Acute tubulo-interstitial nephritis | As principal diagnosis. |
|  | N11 | Chronic tubulo-interstitial nephritis | As principal diagnosis. |
|  | N12 | Tubulo-interstitial nephritis, not specified as acute or chronic | As principal diagnosis. |
|  | N13.6 | Pyonephrosis | As principal diagnosis. |
|  | N15.1 | Renal and perinephric abscess | As principal diagnosis. |
|  | N15.9 | Renal tubulo-interstitial disease, unspecified | As principal diagnosis. |
|  | N28.9 | Disorder of kidney and ureter, unspecified | As principal diagnosis. |
|  | N39.0 | Urinary tract infection, site not specified | As principal diagnosis. |
|  | N39.9 | Disorder or urinary system, unspecified | As principal diagnosis. |
| Perforated/bleeding ulcer | K25.0 | Gastric ulcer, acute with haemorrhage | As principal diagnosis. |
|  | K25.1 | Gastric ulcer, acute with perforation | As principal diagnosis. |
|  | K25.2 | Gastric ulcer, acute with both haemorrhage and perforation | As principal diagnosis. |
|  | K25.4 | Gastric ulcer, chronic or unspecified with haemorrhage | As principal diagnosis. |
|  | K25.5 | Gastric ulcer, chronic or unspecified with perforation | As principal diagnosis. |
|  | K25.6 | Gastric ulcer, chronic or unspecified with both haemorrhage and perforation | As principal diagnosis. |
|  | K26.0 | Duodenal ulcer, acute with haemorrhage | As principal diagnosis. |
|  | K26.1 | Duodenal ulcer, acute with perforation | As principal diagnosis. |
|  | K26.2 | Duodenal ulcer, acute with both haemorrhage and perforation | As principal diagnosis. |
|  | K26.4 | Duodenal ulcer, chronic or unspecified with haemorrhage | As principal diagnosis. |
|  | K26.5 | Duodenal ulcer, chronic or unspecified with perforation | As principal diagnosis. |
|  | K26.6 | Duodenal ulcer, chronic or unspecified with both haemorrhage and perforation | As principal diagnosis. |
|  | K27.0 | Peptic ulcer, site unspecified, acute with haemorrhage | As principal diagnosis. |
|  | K27.1 | Peptic ulcer, site unspecified, acute with perforation | As principal diagnosis. |
|  | K27.2 | Peptic ulcer, site unspecified, acute with both haemorrhage and perforation | As principal diagnosis. |
|  | K27.4 | Peptic ulcer, site unspecified, chronic or unspecified with haemorrhage | As principal diagnosis. |
|  | K27.5 | Peptic ulcer, site unspecified, chronic or unspecified with perforation | As principal diagnosis. |
|  | K27.6 | Peptic ulcer, site unspecified, chronic or unspecified with both haemorrhage and perforation | As principal diagnosis. |
|  | K28.0 | Gastrojejunal ulcer, acute with haemorrhage | As principal diagnosis. |
|  | K28.1 | Gastrojejunal ulcer, acute with perforation | As principal diagnosis. |
|  | K28.2 | Gastrojejunal ulcer, acute with both haemorrhage and perforation | As principal diagnosis. |
|  | K28.4 | Gastrojejunal ulcer, chronic or unspecified with haemorrhage | As principal diagnosis. |
|  | K28.5 | Gastrojejunal ulcer, chronic or unspecified with perforation | As principal diagnosis. |
|  | K28.6 | Gastrojejunal ulcer, chronic or unspecified with both haemorrhage and perforation | As principal diagnosis. |
| Cellulitis | L02 | Cutaneous abscess, furuncle and carbuncle | As principal diagnosis.  Exclude cases with any procedure except those in blocks 1820 to 2016, or if procedure is 30216-00[1604], 30216-01[1604], 30216-02[1604], 30676-00[1659], 30223-01[1606], 30223-02[1606], 30064-00[1605], 90660-00[1602], 90661-00[1608], and this is the only listed procedure. |
|  | L03 | Cellulitis | As principal diagnosis.  Exclude cases with any procedure except those in blocks 1820 to 2016, or if procedure is 30216-00[1604], 30216-01[1604], 30216-02[1604], 30676-00[1659], 30223-01[1606], 30223-02[1606], 30064-00[1605], 90660-00[1602], 90661-00[1608], and this is the only listed procedure. |
|  | L04 | Acute lymphadenitis | As principal diagnosis.  Exclude cases with any procedure except those in blocks 1820 to 2016, or if procedure is 30216-00[1604], 30216-01[1604], 30216-02[1604], 30676-00[1659], 30223-01[1606], 30223-02[1606], 30064-00[1605], 90660-00[1602], 90661-00[1608], and this is the only listed procedure. |
|  | L08 | Other local infections of skin and subcutaneous tissue | As principal diagnosis.  Exclude cases with any procedure except those in blocks 1820 to 2016, or if procedure is 30216-00[1604], 30216-01[1604], 30216-02[1604], 30676-00[1659], 30223-01[1606], 30223-02[1606], 30064-00[1605], 90660-00[1602], 90661-00[1608], and this is the only listed procedure. |
|  | L88 | Pyoderma gangrenosum | As principal diagnosis.  Exclude cases with any procedure except those in blocks 1820 to 2016, or if procedure is 30216-00[1604], 30216-01[1604], 30216-02[1604], 30676-00[1659], 30223-01[1606], 30223-02[1606], 30064-00[1605], 90660-00[1602], 90661-00[1608], and this is the only listed procedure. |
|  | L98.0 | Pyogenic granuloma | As principal diagnosis.  Exclude cases with any procedure except those in blocks 1820 to 2016, or if procedure is 30216-00[1604], 30216-01[1604], 30216-02[1604], 30676-00[1659], 30223-01[1606], 30223-02[1606], 30064-00[1605], 90660-00[1602], 90661-00[1608], and this is the only listed procedure. |
|  | L98.3 | Eosinphilic cellulitis [Wells] | As principal diagnosis.  Exclude cases with any procedure except those in blocks 1820 to 2016, or if procedure is 30216-00[1604], 30216-01[1604], 30216-02[1604], 30676-00[1659], 30223-01[1606], 30223-02[1606], 30064-00[1605], 90660-00[1602], 90661-00[1608], and this is the only listed procedure. |
| Pelvic inflammatory disease | N70 | Salpingitis and oophoritis | As principal diagnosis. |
|  | N73 | Other female pelvic inflammatory diseases | As principal diagnosis. |
|  | N74 | Female pelvic inflammatory disorders in diseases classified elsewhere | As principal diagnosis. |
| Ear, nose and throat infections | H66 | Suppurative and unspecified otitis media | As principal diagnosis. |
|  | J02 | Acute pharyngitis | As principal diagnosis. |
|  | J03 | Acute tonsillitis | As principal diagnosis. |
|  | J06 | Acute upper respiratory infections of multiple and unspecified sites | As principal diagnosis. |
|  | J31.2 | Chronic pharyngitis | As principal diagnosis. |
| Dental conditions | K02 | Dental caries | As principal diagnosis. |
|  | K03 | Other diseases of hard tissues of teeth | As principal diagnosis. |
|  | K04 | Diseases of pulp and periapical tissues | As principal diagnosis. |
|  | K05 | Gingivitis and periodontal diseases | As principal diagnosis. |
|  | K06 | Other disorders of gingiva and edentulous alveolar ridge | As principal diagnosis. |
|  | K08 | Other disorders of teeth and supporting structures | As principal diagnosis. |
|  | K09.8 | Other cysts of oral region, not elsewhere classified | As principal diagnosis. |
|  | K09.9 | Cyst of oral region, unspecified | As principal diagnosis. |
|  | K12 | Stomatitis and related lesions | As principal diagnosis. |
|  | K13 | Other diseases of lip and oral mucosa | As principal diagnosis. |
|  | K14.0 | Glossitis | As principal diagnosis. |
| Convulsions and epilepsy | G40 | Epilepsy | As principal diagnosis. |
|  | G41 | Status epilepticus | As principal diagnosis. |
|  | R56 | Convulsions, not elsewhere classified | As principal diagnosis. |
| Eclampsia | O15 | Eclampsia | As principal diagnosis. |
| Gangrene | R02 | Gangrene, not elsewhere classified | In any diagnosis. |
|  | I70.24 | Atherosclerosis of arteries of extremities with gangrene | As principal diagnosis. |
|  | E09.52 | Intermediate hyperglycaemia with peripheral angiopathy, with gangrene | As principal diagnosis. |
